# Feasibility of Growth Factor Agent Therapy in Repairing Motor Injury

**DOI:** 10.3389/fphar.2022.842775

**Published:** 2022-01-25

**Authors:** Qiaoyin Tan, Jiayu Li, Yuwen Liu, Xiaojuan Zhu, Weide Shao

**Affiliations:** ^1^ College of Teacher Education, Zhejiang Normal University, Jinhua, China; ^2^ College of Physical Education and Health Sciences, Zhejiang Normal University, Jinhua, China; ^3^ Department of General Surgery, The First Affiliated Hospital of Jiangxi Medical College, Shangrao, China

**Keywords:** growth factor, growth factor agent therapy, motor injury, restorative therapy, repair therapy

## Abstract

Growth factors (GF), with the activity of stimulating cell growth, play a significant role in biology, medicine, and exercise physiology. In the process of exercise, human tissues are impacted, making cells suffer damage. Growth factor can accelerate the repair of damaged cells and regulate the synthesis of protein, so biological preparations of growth factors can be added to traditional therapies. A combination of growth factor biologics and conventional therapies may improve the efficiency of injury repair, but growth factor biologics may not produce any results. The feasibility of growth factor biologics in the treatment of motor injury was discussed. The research have shown that: 1) GF biological agent therapy is a very promising treatment for motor injury, which is based on the power of autologous growth factor (GFs) to accelerate tissue healing, promote muscle regeneration, increase angiogenesis, reduce fibrosis, and make the muscle injury rapid recovery. 2) There are various methods for delivering the higher dose of GF to the injured tissue, but most of them depend on the platelet release of GF. At the site of injury, there are several ways to deliver higher doses of GF to the injured tissue. 3) At present, the inhibition of GF is mainly through signal transduction inhibitors and inhibition of transcription factor production. 4) Pattern of GF during wound repair: GF directly regulates many key steps of normal wound repair, including inflammatory cell chemotaxis, division and proliferation of fibroblasts, keratinocytes and vascular endothelial cells, formation of new blood vessels, and synthesis and degradation of intercellular substances. 5) When GF promotes chronic wound healing, in most cases, certain GF can be used targeted only when *in vivo* regulation still cannot meet the need for repair.

## Introduction of Growth Factor

Growth factors, which are cytokines with activity of stimulating cell growth, are composed of polypeptides and protein and are used to regulate cell growth and other cell functions. They are found in platelets, various adult and embryonic tissues, and most cultured cells (Burnouf et al., 2013). It can directly reach the nucleus to repair genes and promote cell division, reproduction and regeneration (Escobar et al., 2015). The general characteristic of GF biological preparations is that it can bind to cell membrane specific receptors and modulate the growth and development of cells. At present, Fibroblast growth factor (FGF), bone morphogenetic protein (BMP), nerve growth factor (NGF), granulocyte colony stimulating factor (G-CSF), platelet-derived growth factor (PDGF), transforming growth factor -α and transforming growth factor -β are all GF related to sports injury. Sports injuries are generally caused by professional sports and recreational sports injuries. Muscle injury usually occurs after eccentric contraction, resulting in injury of tendon joint. When the degree of injury is different, the doctor’s treatment method will also change. But mainly limited to ice compress, oppression, elevation, anti-inflammatory drugs, rest, and activities (Kasemkijwattana et al., 2000). Motor injury generally exists in muscle tissue, bone and other parts of the human body. The spontaneous regeneration ability of muscle tissues and bones is poor, and usually they cannot be fully recovered after injury (Lyu et al., 2020). Once the muscle and bone are damaged, due to the formation of adhesion and scar, the normal biological and biomechanical properties of tendon and bone cannot be completely regained (Giannotti et al., 2015). These abnormal arrangements and structures are often the risk factors for re-injury. Sports-related injuries include acute and chronic injuries. For soft tissues including muscles and ligaments, no matter what kind of injury is, it includes four processes: hemostasis, inflammation, proliferation of cells and matrix, wound maturation and remodeling. The difference lies in the time limit. In the above-mentioned processes, the cell and matrix proliferation stage can be said to be the most important stage of wound healing, determining whether the function of soft tissues is likely to return to the previous level. In addition, the stages of wound maturation and remodeling may involve the formation of scar tissue. Moreover, physiopathologic and metabolic factors in the body can affect any process of injury repair, resulting in prolonged time. In the process of potentially unfavorable repair, it leads to the instability of the soft tissue structure and the weakening of its function, resulting in re-injury. (Middleton et al., 2012)Movement-related GF can affect the cycle conversion of target cells to accelerate the division and proliferation of cells (Creaney and Hamilton, 2008), and promote the synthesis of DNA, RNA and protein in tissue repair cells, which can effectively eliminate low density lipoprotein in blood (Evanson et al., 2014). It plays a significant regulatory part in wound inflammation and infection, wound healing, differentiation of various cells (Gigante et al., 2013), and apoptosis of aging cells in the human body (Bachl et al., 2009).

GF formulations have described a number of fascinating results in sports medicine (Bisciotti et al., 2013), however, the biological characteristics of many different GF preparations are not well understood. A variety of GF preparations can be used in combination, which makes the effect better in the repair of sports injury. However, the effect of using GF preparation in the treatment of motor injury may also be poor, so we must pay attention to the implementation of the course of treatment and the role of the site. In this paper, the source, properties, action mechanism and possible effects of GF preparations in the field of sports injury were introduced in detail. The course results of future GF preparations for the treatment of sport injury were also summarized in order to promote the development of GF preparation therapy.

## Performance of Growth Factor

Bone morphogenetic protein-2 and PDGF are the barrier membranes of osteogenic growth factors, and they have studied the loading and release kinetics of growth factors more through soaking in GF solution and culturing the membrane followed by freeze-drying, or mixing with polymer before evaporation to incorporate GF into the membrane (Zumstein et al., 2011). Membrane materials and other reagents are important factors that affect the adsorption capacity of the membrane, as well as the concentration of heparin, crosslinking agent and GF. Notably, there were two stages of release of GF from the membrane: the first stage consisted of a sudden release (approximately 1 day) and the second stage consisted of a slow release. In addition, the ability to exert biological activity at GFs concentrations controlled by release in the membrane has also been demonstrated by many studies. For example, white blood cell and platelet-rich fibrin (L-PRF) contains growth factors. L-PRF clots were slowly released with increasing GF1, PDGF-AB over the first 8 h, and within the first 7 days increased complete release of growth factors GF1, VEGF, and MPO and then decreased to near zero at Day 28.

Studies have shown that there are several methods for delivering higher doses of GF to injured tissues, but most rely on platelet release of GF. The alpha particles of platelets contain GF [insulin-like GF-1, vascular endothelial GF, and transformed GF-β (1)],etc. Platelet-rich plasma is usually analyzed for peripheral blood using different types of centrifugation, and different separation methods result in different distributions of platelet and leukocyte counts (Pochini et al., 2016). A standard L-PRF centrifugation protocol at 400 ×g has been shown to have the highest concentrations of platelets and leukocytes (Zumstein et al., 2012).

**FIGURE 1:**
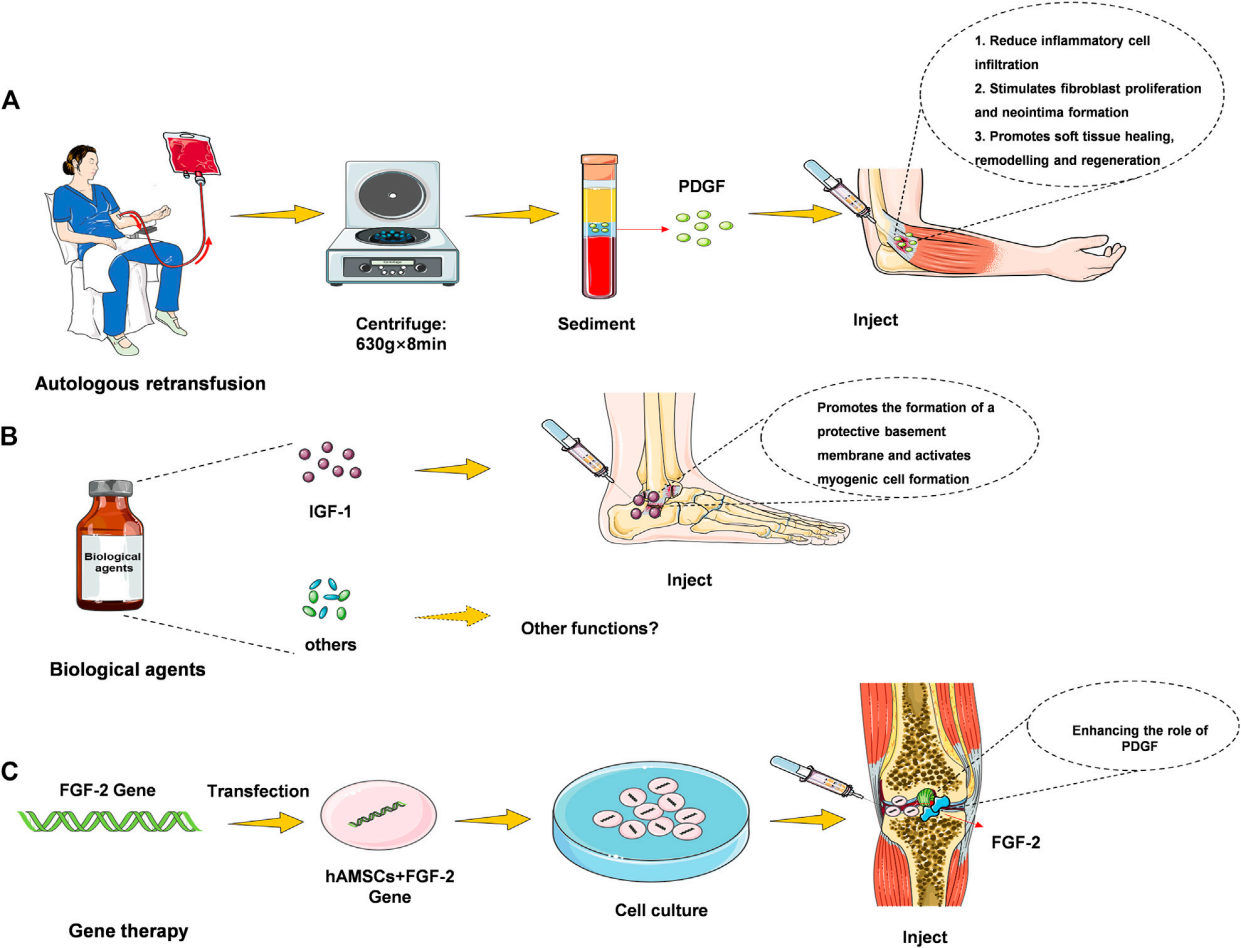
Feasibility of growth factor preparation in the repair of sports injury.

Plasma (PRGF) therapy, direct injection and hydrogel incorporation can be realized at the injury site as can be seen in Figure 1. 1) Plasma treatment refers to the reinfusion of plasma in the body. After removing red and white blood cell, platelets, and liquid components from that blood, the remaining material contain large amounts of growth factors, coagulation factors, albumin, globulin, and various antibodies. 2) Direct injection, which can be used as a liquid injection preparation for damaging tendons or joints (Singh et al., 2020). Pure platelet-rich fibrin (P-PRF) and leukocyte and platelet-rich fibrin (L-PRF) are leukocyte-free and leukocyte-containing solid fibrin-based products, respectively, that can be used as bioactive healing biomaterials (Creaney et al., 2011). 3) Hydrogel therapy. Treatment of motor injury requires consistent skeletal muscle regeneration, complete healing as far as possible, and reduction of tissue fibrosis (Hwang et al., 2013). It has been studied that stem cells and basic fibroblast growth factor (BFGF) are incorporated into hydrogels to observe muscle cell regeneration in animal models of muscle tearing (Caballé-Serrano et al., 2019). The results showed that the rapid twitch muscle contraction at the injury site was markedly improved, and fibrosis was markedly reduced. Combined treatment with GF hydrogel results in restoration of function, revascularization and innervation with minimal fibrosis of the torn muscle. Thus, some of the GF hydrogel preparations could be used as a hopeful treatment for muscle cells’ regeneration, and this part of the study needs to be further developed.

In terms of the effect of activator on promoting the use of GF, some studies have found that the activator used in combination with PRP is effective for promoting the concentration of GF. Such as the effect of activator calcium chloride on the concentration of GF, activated and inactive calcium chloride prepared by PRP under each condition, activated with calcium chloride resulted in significant increases in PDGF antibody and IGF-1 concentrations, and calcium chloride activated PRP resulted in the release of platelet-differentiated GF (Hamilton et al., 2015). The use of these activators affects the outcome after treatment of motor injury and is recommended for consideration in future treatment.

## Inhibition of Growth Factor

GF is the key to regulate cell signaling pathway. Currently, the inhibition of GF is exerted through signal transduction inhibitors. It can interfere with the process of protein-protein interaction between GF and its receptor by targeting the extracellular protein-binding domain of GF receptor. Such as the signal transduction inhibitor compound CJJ300, inhibits tgf-beta signals by disrupting the process of formation of signal complexes that transform gf-beta (tgf-beta) (Wu et al., 2020). The compounds can be used for treating the elimination of downstream signals during the transmission of cell signaling pathways, the phosphorylation of GF in key factors of the pathways and the induction of molecular markers. Through these effects, signaling inhibitors can significantly inhibit cell migration.

In addition, there is a method for producing an inhibitory effect on GF by inhibition of a transcription factor. Mechanical GF (MGF) can significantly promote the migration of chondrocytes from growth plates, accompanied by YAP activation. YAP is a transcription regulator that modulate cell proliferation, survival, and differentiation, in order to control organ development and tissue regeneration (Jing et al., 2018). Knocking out YAP with the YAP inhibitor YAP siRNA inhibits MGF-induced migration. MGF promotes YAP activation through RhoA GTPase-mediated cytoskeletal recombination, and C3 toxin can also be used to inhibit RhoA and eliminate MGF-induced YAP activation.

With the development of GF biological agents, many studies began to study GF inhibitors to achieve the purpose of inhibiting GF and controlling its action mechanism.

**TABLE 1:** Therapeutic use of growth factor preparation.

Growth factor	Extraction method	Save method	Disposal method	Treatment site	Treatment	Biological effect	Mechanism of action	References
Plasma rich in growth factors (PRGF)	630 g centrifuge 8 min to get the middle layer	stored at −80°C	10% calcium chloride activation	Achilles tendon of sheep injury	Infiltration injection	Regulating the inflammatory reaction of the achilles tendon injury, accelerating the tendon healing process and shortening the recovery time	Reduce tendon inflammatory cell infiltration	Daniel Aguilar-García et al.(2018) (Aguilar-Garcia et al. (2018)
Fibroblast growth factor (FGF-2)	NM	NM	HAMSCs were transfected with a lentivirus carrying the FGF-2 gene and combined with autologous platelet-rich plasma (PRP)	Rabbit bone-tendon interface	Local injection	HAMSCs transfected with FGF-2 gene in combination with autologous PRP can enhance tendon-to-bone healing	FGF-2 and PRP have synergistic effect	Jun Zhang et al.(2020) (Zhang et al. (2020)
(PRGF-Endoret)	460 g centrifuge 8 min to get the middle layer	stored at −80°C	10% calcium chloride activation	Medial collateral ligament of rabbit	Local injection	Local application of PRGF-Endoret advances early pace of ligament healing and recovery of structural features in rabbit models	PRGF-Endoret stimulates fibroblast proliferation and neovascularization	Tomokazu Yoshioka et al.(2013) (Yoshioka et al. (2013)
Insulin-like growth factor 1 (IGF-1)	NM	NM	NM	NM	Local injection	Exogenous insulin-like growth factor -1 accelerates the process of skeletal muscle post-traumatic repair induced by treadmill exercise	It can promote the formation of protective film of basement membrane, activate myoblasts to form myofilaments and myotubes, and promote fusion into muscle fibers	Wang, Qing et al.(2021) (Wang and Wang (2021)
Bevacizumab, an anti-vascular endothelial growth factor (VEGF) preparation	NM	NM	NM	Knee joint of rat with chronic athletic arthritis injury	Intra-articular injection	Bevacizumab Treatment Improves Cartilage Degradation in Rats Suffering from Chronic Exercise-induced Arthritis Injury	Bevacizumab treatment resulted in marked decreases in cytokine interleukin (IL)-1β, tumor necrosis factor (TNF)-α, matrix metalloproteinase (MMP)-1 and MMP-3 levels, and marked increases in transforming growth factor (TGF)-β1 levels	Lei Shang et al.(2018) (Shang et al. (2018)
(PRGF-Endoret)	460 g centrifuge 8 min to get the middle layer	stored at −80°C	10% calcium chloride activation	Knee joint with anterior cruciate ligament (ACL) tear	Intra-articular injection	PRGF-Endoret Promotes Exercise Recovery in Athletes with Cases of Instability Caused by ACL Tears	NM	Roberto Seijas et al.(2014) (Seijas et al. (2014)
(PRGF)	460 g centrifuge 8min to get the middle layer	stored at −80°C	10% calcium chloride activation	Knee joint cartilage of young football player	Intra-articular injection	Promote that complete healing of the articular cartilage to be obviously accelerate	NM	Mikel Sánchez et al.(2003) (Mikel Sanchez et al., 2003)
(PRGF)	460 g centrifuge 8 min to get the middle layer	stored at −80°C	10% calcium chloride activation	Ankle joint of player with anterior tibiofibular ligament (AITFL) injury	Intra-articular injection	Combined joint re-stabilization with less long-term residual pain	NM	Lior Laver et al.(2015) (Laver et al. (2015)
(PRGF)	460 g centrifuge 8 min to get the middle layer	stored at −80°C	10% calcium chloride activation	Type III injured ankle ligament	Intra-articular injection	Lower the ankle to show signs of instability and return to previous athletic activity	The entry of growth factors into the injured tissue promotes the healing and remodeling of the soft tissues for regeneration. At lower interleukin levels, the inflammatory healing phase is inhibited, pain is alleviated, and the process of repair and regeneration is accelerated	R Frei et al.(2008) (Frei et al. (2008)

## The Effects of Growth Factor Preparation

### Positive Impact

Pattern of GF in the process of wound repair: GF directly regulates many key steps of normal wound repair, including inflammatory cell tropism, division and proliferation of fibroblasts, keratinocytes and vascular endothelial cells, formation of new blood vessels, and synthesis and degradation of intercellular substance (Mishra et al., 2012). The presence of GF contributes to the healing and remodeling of the soft tissues, and regeneration can begin before leukocytes infiltrate the affected sites. At low interleukin levels, the inflammatory phase of healing is inhibited, pain is alleviated, and the process of repair and regeneration is accelerated (Sanchez et al., 2009). GF mimics the clotting physiological events of thrombin-induced fibrin formation and platelet activation and has the key advantage of not having tissue necrosis effects and being biodegradable by body enzymes. Clinically, most of the platelet-rich GF biomaterials are used to achieve wound healing (Seijas et al., 2014), repair soft and hard tissues (Scala et al., 2014), and stimulate bone tissue regeneration (Zhang et al., 2020). They have recently been evaluated in the repair of knee osteoarthritis and muscle damage (Huang et al., 2020), and platelet GF opens up new prospects for regenerative medicine.

When being applied to the repair of articular cartilage injury and meniscus injury, the patients can move more smoothly when being injected with the GF, and the discomfort is obviously relieved (Shang et al., 2018); and the method is quite effective for treating pain of patients with osteoarthritis. In the area between the pits and the fixed fragments injected with a number of growth factors in autologous plasma, in the case with a poor prognosis, the complete healing of the articular cartilage was significantly accelerated, the functional results were good, and the asymptomatic motor activity could be quickly restored (Sanchez et al., 2003). When being applied to the injury of anterior cruciate ligament and achilles tendon, the GF biological preparation promotes the process of “ligament ingrowth, creeping substitution and bone tendon healing” of the injured ligament (Rizzello et al., 2012) and the surgically reconstructed ligament (Anitua et al., 2014). Studies have found that bone marrow mesenchymal stem cells transfected with FGF-2 gene compound with autologous PRP transplantation can promote extraarticular tendon-bone healing, particularly for acute exercise-related tendon-ligament injury (Laver et al., 2015). The use of PRGF for fear of partial anterior cruciate ligament tears, with the preservation of an intact bundle, can guarantee a high success rate in the recovery from the motor injury (Volpi et al., 2010; Yoshioka et al., 2013).

It has more obvious effect when being used for the treatment of “heel pain” of plantar fasciitis compared with the “local sealing treatment”. Local injection of exogenous insulin-like growth factor-1 (IGF-1) can stimulate the proliferation of myoblasts and accelerate the process of repair (Wang and Wang, 2021). It can be used for the treatment of shoulder arthritis (subacromial and bilateral tendonitis), traumatic or degenerative joint swelling and synovitis, and helps to relieve swelling and pain. The level of evidence is determined by the number of high-quality studies with consistent findings. Patients with ankylosing spondylitis used in the knee joint and sacroiliac joint, have achieved good curative effect, in addition to pain relief, laboratory indicators of erythrocyte sedimentation rate and C-reactive protein also fell to the normal range after 1 month of treatment (Aguilar-Garcia et al., 2018). Regarding the treatment of tendonitis and tenosynovitis, for patients who are resistant to first-line physical therapy such as eccentric load, ABI or PRP injection is an effective second-line treatment to improve clinical results (Sandrey, 2014). The GF biological preparation can also be applied to the treatment of shoulder, elbow, wrist, hip, knee, ankle, sacroiliac joint, vertebral joint and the like (Frei et al., 2008).

### Negative Effect

1) The surrounding environment of wound healing contains protein GF. Its content changes during the whole release process. When the effect of GF is inhibited, wound repair will encounter obstacles (Shen et al., 2021). 2) The surrounding environment of chronic trauma contains low GF activity. 3) When GF promotes the healing of chronic wounds, in most cases, exogenous GF does not necessarily need to be supplied for wound repair, and only when *in vivo* regulation still cannot meet the need for repair, such as some hard-to-heal injuries, can certain GF be used pointedly. For example, local application of IGF-1 in human body has been proved to rapidly stimulate tendon collagen synthesis. Then can IGF-1 injection enhance tendon synthesis and tissue structure in patients with patellar tendon disease? Although clinical responses were small and rapid with IGF-1 injection in combination with training, no additional long-term effects of intratendon IGF-1 on structure and clinical outcome were observed in patients with patellar tendon disease (Olesen et al., 2021). As another example of meniscus injury, the local application of vascular growth factor, vascular endothelial growth factor could theoretically promote healing of the meniscus tear in the avascular zone, however, no meniscus healed completely in this treatment group. It was found that topical application of vascular endothelial growth factor through PDLLA-coated sutures did not promote meniscus healing (Petersen et al., 2007).

Therefore, GF may not always be a hopeful tool for repairing damage, which is related to the site of sports injury, the design of treatment, the number and type of growth factor injections, and further research is needed to clarify the ineffective mechanism.

## Conclusion and Prospect

Muscle damage is a common cause of long-term severe pain and limb disability. Recovery from muscle damage caused directly or indirectly by exercise is a complex but well-defined process that includes degeneration, inflammation, regeneration, and fibrosis. Biological agents, such as IGF-1, MGF, PDGF, and TGF-β have been discussed in clinical treatment. They have the potential not only to treat athletic injuries but also to be a centre of interest for stimulant abusers as biotechnologies that achieve good athletic performance but are not yet fully understood.

The research have shown that: 1) GF biological agent therapy is a very promising treatment for motor injury, which is based on the power of autologous growth factor (GFs) to accelerate tissue healing, promote muscle regeneration, increase angiogenesis, reduce fibrosis, and make the muscle injury rapid recovery. 2) There are various methods for delivering the higher dose of GF to the injured tissue, but most of them depend on the platelet release of GF. At the site of injury, there are several ways to deliver higher doses of GF to the injured tissue. 3) At present, the inhibition of GF is mainly through signal transduction inhibitors and inhibition of transcription factor production. 4) Pattern of GF during wound repair: GF directly regulates many key steps of normal wound repair, including inflammatory cell chemotaxis, division and proliferation of fibroblasts, keratinocytes and vascular endothelial cells, formation of new blood vessels, and synthesis and degradation of intercellular substances. 5) When GF promotes chronic wound healing, in most cases, certain GF can be used targeted only when *in vivo* regulation still cannot meet the need for repair.

To date, most treatments for muscle injury with GF biologics have been due to limited experimental and clinical data or only empirical trials. Therefore, we need more experimental studies, including the specific GFs to quantify the release of PRP, angiogenesis, myogenesis and functional recovery data, as well as other mechanisms of GF repair, to finally verify the repair hypothesis of GF biological agents in the treatment of muscle injury and open a new journey for its wide clinical application. Currently, there is a large amount of data on the pathophysiology of muscle injury in the relevant studies.
